# What Drags the Life out of a Woman

**Published:** 1889-07

**Authors:** 


### WHAT DRAGS THE LIFE OUT OF A WOMAN.

Those heavy skirts, varying in number from three to seven or more, all suspended from the waist and pulling down upon the hips, are enough to drag the life out of a Hercules. A strong man would not endure for a single day one-tenth of the discomfort which a fashionable woman suffers every day of her life. It is useless for woman to think of rising above her present level while she is chained down by the burdens imposed by heavy, trailing skirts.

The unnecessary and injurious weight occasioned by superfluous length and number of skirts is greatly increased by the addition upon the outer garment of an indefinite number of flounces, folds, heavy overskirts and various other useless accessories.

But the evils and inconveniences above referred to are not the worst which result from the wearing of so great a weight of clothing as is customary among fashionable people. The most serious consequences are those which are suffered by the delicate internal organs. The many heavy skirts and under-garments which are hung about the waist with no support from above, drag down the organs of the abdomen, and after a time the slender ligaments which hold them in place give way, and various kinds of displacements and other derangements occur. The tightness with which the garments are drawn at the waist greatly increases the injury.

The custom of wearing the pantaloons buttoned tightly at the top and sustained by the hips, produced so much disease even among the hardy soldiers of the Russian army, that a law was enacted making the wearing of suspenders compulsory. If strong men suffer thus, how much greater must be the injury to frail, delicate women? The constant pressure and unnatural heat to which the lower part of the back is subjected, is one of the chief causes of the frequency of kidney diseases among women. Here is found the source of "weak back," lumbago, pain in the side, and several other diseases of the trunk which affect so many thousands of American women.—J. H. K., in *Dress.*

